# Effect of supplying a portion of trace mineral amino acid complexes on serum folate concentration from the dry period to early lactation

**DOI:** 10.3168/jdsc.2025-0833

**Published:** 2025-10-31

**Authors:** M. Duplessis, A.L. Kerwin, D.H. Kleinschmit, M.T. Socha, T.R. Overton

**Affiliations:** 1 Centre de recherche et développement de Sherbrooke, Agriculture and Agri-Food Canada, Sherbrooke, QC, J1M 0C8, Canada; 2 Department of Animal Science, Cornell University, Ithaca, NY 14853; 3 Zinpro Corporation, Eden Prairie, MN 55344

## Abstract

Folates are required in one-carbon metabolism and DNA methylation. During the transition period, these roles are particularly important for the final stages of fetal development and the onset of lactation. The objective of this retrospective study was to assess plasma folate concentration when a portion of dietary inorganic chloride trace minerals (ITM) and cobalt (Co) carbonate was replaced by AA complexes of trace minerals (AATM) and Co glucoheptonate during the transition period. Relationships between serum folate concentration and health biomarkers were also evaluated. With dietary trace mineral concentrations being the same between treatments, a total of 36 Holstein multiparous cows were randomly assigned based on their parity number and previous 305-d mature equivalent milk production to either (1) an ITM blend consisting of zinc (75 mg/kg DM), manganese (65 mg/kg), and copper (10 mg/kg) as hydroxychlorides, and cobalt (1 mg/kg) as carbonate (ITM) or (2) partial replacement of ITM with AATM of Zn (40 mg/kg), Mn (20 mg/kg), Cu (3.5 mg/kg), and Co glucoheptonate (1 mg/kg; AATM, Availa-Dairy, Zinpro Corp.) from 1 wk after dry-off through 8 wk of lactation. Milk yields were recorded at each milking. Blood samples were taken at wk −8 (before treatment administration), −1, 1, 2, and 8 relative to parturition for serum folate analysis. Serum folate concentration from wk −1 to 8 relative to parturition averaged 8.39 (SE: 0.44) ng/mL and was not affected by treatments. Regardless of treatments, serum folate concentration significantly decreased from wk −8 to −1, plateaued from wk −1 to 2 relative to parturition, and then significantly increased through wk 8 of lactation. Milk yield, DMI, serum alanine aminotransferase, and alkaline phosphatase concentrations were positively correlated with serum folate concentration, whereas serum BHB concentration tended to be negatively correlated with serum folate concentration. In conclusion, in the current study, the source of the trace mineral supplement fed during the dry period and early lactation did not have an impact on serum folate concentrations.

## Highlights

• Milk yield tended to increase by 6.6% with amino acid complexes of trace minerals.
• Serum folates were not affected by the source of the trace mineral supplement.
• Serum folates varied during the dry period and the onset of lactation.
• Milk yield and dry matter intake were positively correlated with serum folate.

## Graphical Abstract

**Figure:**
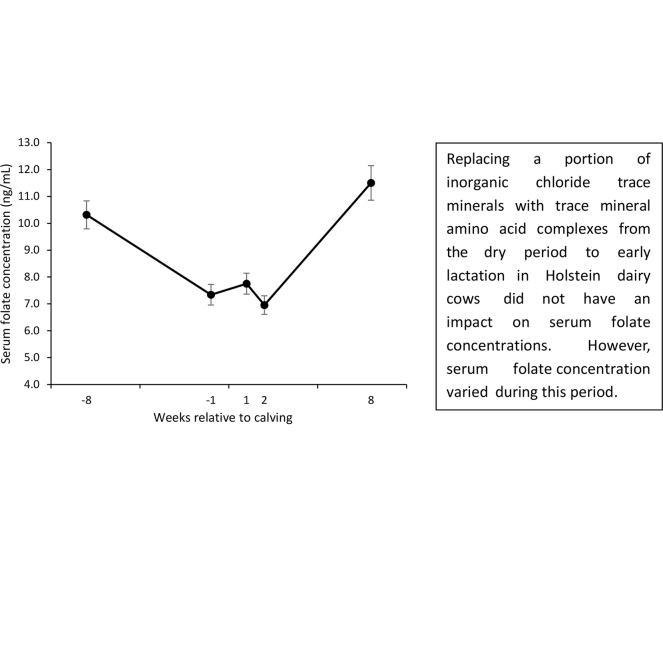
**Summary:** A total of 36 pregnant Holstein cows received either (1) an inorganic chloride trace mineral blend consisting of zinc (75 mg/kg of dry matter), manganese (65 mg/kg), and copper (10 mg/kg) as hydroxychlorides and cobalt (1 mg/kg) as carbonate or (2) partial replacement of inorganic trace minerals with amino acid complexes of trace mineral source of zinc (40 mg/kg), manganese (20 mg/kg), copper (3.5 mg/kg), and cobalt glucoheptonate (1 mg/kg) from 1 week after dry-off through 8 weeks of lactation. Serum folate concentration from week -1 to 8 relative to parturition averaged 8.39 (standard error: 0.44) ng/mL and was not affected by treatments. Milk yield, dry matter intake, serum alanine aminotransferase, and alkaline phosphatase concentrations were positively correlated with serum folate concentration, whereas serum β-hydroxybutyrate concentration tended to be negatively correlated with serum folate concentration.

During the transition period, cows face several physiological and immune changes. Activation of the immune system is observed in almost all periparturient dairy cows when adapting to this new physiological stage (Horst et al., 2021). This is also a period in which cows are more at risk of metabolic disorders diminishing cow productivity and longevity. Nutrition plays a critical role in helping cows to cope with this challenging period. For instance, trace minerals such as Cu, Mn, and Zn are needed in small amounts in the diet but have important roles as enzyme components in immune and oxidative systems (Overton and Yasui, 2014). For example, Zn is ubiquitous in the body and has a role in the methylation cycle (McGee et al., 2018). Cobalt is required for vitamin B_12_ synthesis by ruminal bacteria (Martens et al., 2002). In a meta-analysis, it has been reported that organic trace mineral supplementation increased milk yield compared with inorganic trace mineral supplement although there was variability among studies (Rabiee et al., 2010). It was hypothesized that this was due to improved bioavailability of an organic trace mineral supplement versus an inorganic trace mineral supplement (Kerwin et al., 2023).

In mammals, folates, which refer to compounds exhibiting the biological activity of folic acid, are required in several important metabolic and physiological functions such as one-carbon metabolism, DNA methylation, erythropoiesis, and immune function (Combs and McClung, 2022). Because of the extensive degradation of preformed labile methyl groups in the rumen, ruminants largely rely on de novo synthesis of methyl groups via the one-carbon pool, which depends on adequate supply of folates (McFadden et al., 2020). Indeed, the methylated form of folates can give its methyl group to homocysteine to form Met, which can then be used in protein synthesis or transformed to S-adenosylmethionine (McFadden et al., 2020). The latter is a major donor of labile methyl groups used in over 100 enzymatic reactions (Combs and McClung, 2022). Milk production increases the demand of methyl groups as milk contains high amounts of methyl compounds (Xue and Snoswell, 1985). In the last decades, some studies reported that a folic acid supplement, given alone or in combination with a vitamin B_12_ supplement around parturition, increased milk yield in early lactating cows (Girard and Duplessis, 2023), especially when plasma folate concentrations of cows were below 12 ng/mL (Girard and Graulet, 2021). Thus, it suggested that, particularly at the onset of lactation, folate supplies from ruminal synthesis and the diet were not sufficient to optimize animal performance and health. It is also known that dietary factors such as fiber and nonfiber carbohydrate concentrations can respectively negatively and positively influence plasma folate status (Duplessis et al., 2020), probably by affecting ruminal synthesis of the vitamin. It has been suggested that the source of trace mineral supplement has an impact on digestion and ruminal fermentation (Guimaraes et al., 2022) and then can influence ruminal synthesis of folates. However, the impact of the source of the trace mineral supplement on serum folate concentrations as an indicator of the vitamin status has been barely studied. The objective of this study was to assess serum folate concentrations when a portion of dietary inorganic chloride trace minerals (**ITM**) and Co carbonate was replaced by AA complexes of trace minerals (**AATM**) and Co glucoheptonate. In addition, relationships were assessed between serum folate concentration and milk yield, DMI, serum liver function biomarker concentrations, and the liver health index.

All procedures on animals were approved by the Cornell University Institutional Animal Care and Use Committee (#2019–0037). The present retrospective study used a convenient cow subset from the experiment of Kerwin et al. (2023). A total of 36 pregnant Holstein cows (out of 69 cows) entering their second or greater lactation (16 cows entering their second and 20 entering their third or greater lactations) were randomly chosen for the purpose of the current study. At dry-off, cows were moved to tiestalls and fed the far-off diet without any treatment for a week (between d 64 and 57 before expected calving date). Based on their parity group and previous 305-d mature equivalent milk production, cows were then randomly assigned to one of the following treatments using JMP Pro (version 14, SAS Institute Inc., Cary, NC): (1) an inorganic trace mineral blend consisting of Zn (75 mg/kg DM), Mn (65 mg/kg), and Cu (10 mg/kg) as hydroxychlorides and Co (1 mg/kg) as carbonate (ITM) or (2) partial replacement of inorganic trace minerals with AA complexes of Zn (40 mg/kg), Mn (20 mg/kg), and Cu (3.5 mg/kg), and Co from Co glucoheptonate (1 mg/kg; AATM, Availa-Dairy, Zinpro Corp., Eden Prairie, MN). Treatments are based on what can be typically encountered on farms and based on the study by Nocek et al. (2006). The farm staff was blinded to treatment, but the research crew was not blinded to treatment allocation. Treatments were given from 1 wk after dry-off through 8 wk of lactation. Dietary ingredient inclusions and total dietary supplies from the trace mineral supplements were kept the same between treatments. All diets were formulated with the Cornell Net Carbohydrate and Protein System (v. 6.5.5, Cornell University, Ithaca, NY) and were detailed by Kerwin et al. (2023). Briefly, far-off diets fed from dry-off to d 21 before expected parturition included 40.2% of DM of corn silage, 34.6% of DM of wheat straw, and 25.2% of DM of concentrate; close-up diets fed from d 21 before expected calving to parturition had 43.3% of DM of corn silage, 30.0% of DM of wheat straw, and 26.7% of DM of concentrate; and lactation diets fed after parturition had 45.0% of DM of corn silage, 16.9% of DM of hay crop silage, and 38.1% of DM of concentrate, regardless of treatments. The ITM supplement contained calcium carbonate (67.9%), Intellibond M (Mn, 14.8%; Selko, Indianapolis, IN), Intellibond Z (Zn, 13.7%; Selko), Intellibond C (Cu, 1.8%; Selko), mineral oil (1.5%), cobalt carbonate (0.2%), ethylenediamine dihydroiodide (0.2%), and sodium selenite (0.1%), delivering 75,075 mg/kg Zn, 65,120 mg/kg Mn, 10,150 mg/kg Cu, 1,023 mg/kg Co, 1,200 mg/kg I, and 293 mg/kg Se (DM basis). The AATM supplement contained Availa-Dairy (60.0%), calcium carbonate (20.6%), Intellibond M (10.2%; Selko), Intellibond Z (6.4%; Selko), Intellibond C (1.1%; Selko), mineral oil (1.5%), ethylenediamine dihydroiodide (0.2%), and sodium selenite (0.1%), delivering 75,000 mg/kg Zn (53.4% organic Zn), 64,920 mg/kg Mn (30.9% organic Mn), 10,060 mg/kg Cu (35.1% organic Cu), 1,002 mg/kg Co (100% organic Co), 1,200 mg/kg I, and 293 mg/kg Se. Trace mineral supplements were given at 0.1% of DM in all diets, mixed with corn distillers ethanol (1.0% of DM), and then blended with the whole diets. Throughout the experimental period, cows were fed once daily between 0900 and 1100 h and between 0700 and 0900 h for prepartum and postpartum diets, respectively, and daily intake was measured by recording the amount of TMR offered and refused. Samples of TMR were taken weekly, dried in a forced-air oven for 96 h at 48.5°C, composited at 4-wk intervals, and ground at 2 mm using a Wiley Mill (Arthur H. Thomas, Swedesboro, NJ). Folate concentrations of far-off (only samples without treatment inclusion), close-up, and lactation diets were analyzed as described by Beaudet et al. (2016) using a commercial microbiological microtiter plate test (Vita-Fast Folic Acid, R-Biopharm Inc., Marshall, MI). The interassay CV was below 5%.

Body weight and BCS based on a 1-to-5 scale with quarter points (Edmonson et al., 1989) were obtained before treatment assignment and at wk −1, 1, 2, and 8 relative to expected calving date. Cows were milked thrice daily, and milk yields were recorded at each milking by an on-farm milk recording system (Delpro, DeLaval, Gurnee, IL). Blood samples were taken before feeding between 0600 and 0730 h by tail venipuncture using one 10-mL plain tube with a Vacutainer system (Becton, Dickinson and Co., Franklin Lakes, NJ) before treatment assignment and at wk −1, 1, 2, and 8 relative to expected parturition date. Blood samples were centrifuged at 1,400 × *g* for 20 min at 4°C after a standing time of at least 30 min, allowing blood to clot at room temperature. Serum folate concentration was analyzed in triplicate according to the manufacturer instructions (Folate/Vitamin B_12_ ChLIA Kit, MP Biomedicals, Solon, OH). The main form of folates found in serum is 5-methyl-tetrahydrofolic acid. The interassay CV was 7.8%. At wk 1 and 2, serum BHB (colorimetric kit, Catachem Inc., Oxford, CT), haptoglobin (colorimetric kit, Tri-Delta Diagnostics Inc., Boonton Township, NJ), urea, creatinine, albumin, total protein, globulin, cholesterol, bilirubin, alanine aminotransferase (**ALT**), alkaline phosphatase (**ALP**), and gamma-glutamyl transferase (automated photometric analyzer, University of Missouri Veterinary Medical Diagnostic Laboratory, Columbia, MO) were analyzed, as detailed by Kerwin et al. (2023). The liver health index was calculated from serum variables obtained at wk 1 and 2 for the current subset of cows as follows: [(albumin − µ_albumin_)/σ_albumin_] + [(cholesterol − µ_cholesterol_)/σ_cholesterol_] − [(bilirubin − µ_bilirubin_)/σ_bilirubin_], where µ = the overall sampling population mean and σ = the overall sampling population SD (Kerwin et al., 2022).

Weekly milk yields and DMI were obtained by averaging daily data at wk 1, 2, and 8 relative to parturition. All statistical analyses were conducted using SAS software (version 9.4, SAS Institute Inc.). Weekly DMI was also calculated for the week before treatment assignment and at wk −1 relative to calving. Data for DMI, BW, BCS, and serum folate concentration obtained the week before the start of the treatment period and dietary concentrations of folates in the close-up and lactation periods were analyzed by ANOVA using Proc MIXED with treatment groups (ITM or AATM) as fixed effect. Serum folate concentrations, DMI, BW, BCS, and milk yield over sampling times (wk −1, 1, 2, and 8 relative to parturition, except for milk yield wk 1, 2, and 8 relative to parturition) were assessed by ANOVA in Proc MIXED using repeated measurements with treatment group, week, and the interaction were fixed effects. Moreover, except for milk yield, each model had a respective covariate value that corresponded to data obtained before treatment assignment included as a fixed effect. As samples were not evenly spaced, the following covariance structures were alternatively tried: spatial power, exponential spatial power, spatial Gaussian, spatial linear, spatial linear log, spatial spherical, first-order antedependence, and unstructured. The one leading to the smallest Bayesian information criterion was retained. Similarly, ANOVA models with unevenly spaced repeated measurements using Proc MIXED and solely time as a fixed effect were used to evaluate serum folate concentrations according to all sampling points regardless of treatments. A Tukey test was applied when the time effect was significant. Normality and homoscedasticity were visually assessed by the quantile-quantile plot and the studentized residuals by predicted value plot, respectively. The MEANS procedure was used to obtain averages and SD of actual DIM at sampling times, lactation numbers, prepartum days on treatments for each treatment group, and folate concentrations of the far-off diet before treatment inclusion. Spearman rank correlation coefficients were obtained using Proc CORR between serum folate concentrations and serum biomarkers and the liver health index at wk 1 and 2 due to non-normality detected by visual assessment of graphs from Proc UNIVARIATE of some biomarkers. Correlations between serum folate concentrations and milk yield (wk 1, 2, and 8) and DMI (wk −1, 1, 2, and 8 relative to calving) were evaluated using Proc CORR. Results were considered significant when *P* ≤ 0.05 and a tendency when 0.05 < *P* ≤ 0.10.

On average, cows had 2.9 (SD: 0.9) lactations after calving and received their treatment for 52 (SD: 4) d before parturition. Actual days relative to parturition for sample collection were −56 (SD: 4; covariate sample), −4 (SD: 2; wk −1 sample), 5 (SD: 1; wk 1 sample), 12 (SD: 1; wk 2 sample), and 53 (SD: 2; wk 8 sample) DIM. Far-off dietary folate concentrations before treatment inclusion were 0.32 (SD: 0.06) mg/kg DM. Previous lactation 305-d mature equivalent milk production did not differ between treatments and averaged 14,340 (SE: 387) kg (*P* = 0.99). Before treatment assignment, DMI and BW were greater for cows fed AATM than ITM by 1.4 kg/d and 63 kg, respectively (*P* < 0.05). At enrollment, BCS averaged 3.38 (SE: 0.07; *P* = 0.19). Before treatment assignment, at about wk −8 before calving, serum folate concentrations were 10.32 (SE: 0.75) ng/mL in both treatment groups (*P* = 0.86), which was lower than previously reported using a different laboratory method (Girard et al., 1989).

Folate concentrations of close-up (0.28 [SE: 0.01] mg/kg DM; n = 8) and early-lactation (0.35 [SE: 0.02] mg/kg DM; n = 26) diets did not differ between treatments (*P* > 0.13). Compared with other values reported in the literature (Duplessis et al., 2020; Brisson et al., 2022), averaged dietary folate concentrations were lower in the current study. Indeed, in a cross-sectional study conducted in North America, averaged folate concentrations of lactating diets ranged from 0.43 to 1.38 mg/kg DM (Duplessis et al., 2020). Moreover, in a meta-analysis including 15 studies, averaged dietary folate concentration for lactating cows was 0.50 (SD: 0.21) mg/kg DM (Brisson et al., 2022).

There was no treatment effect on DMI, BW, and BCS from wk −1 to 8 relative to calving (*P* > 0.37; Table 1). Milk yield tended (*P* = 0.07) to increase by 6.6% for AATM groups within the first 8 wk of lactation (Table 1). These results are similar to the ones reported by Kerwin et al. (2023), as the current results were obtained from a subset of cows from this last study. Kerwin et al. (2023) hypothesized that this could be due to an increased trace mineral availability and an improved immune function in early lactation.

**Table 1:** Averaged DMI, BW, BCS, milk yield, and serum folate concentrations at wk −1, 1, 2, and 8 relative to parturition of cows fed different sources of trace minerals

Item	Treatment^1^		SEM	*P*-value	
	ITM	AATM		Treatment	T × wk^2^
Cows (n)	18	18			
DMI^3^ (kg/d)	17.9	18.4	0.4	0.37	0.48
BW^3^ (kg)	745	747	6	0.86	0.45
BCS^3^	3.27	3.28	0.03	0.81	0.68
Milk yield^3^ (kg/d)	42.2	45.0	1.0	0.07	0.89
Serum folates^3^ (ng/mL)	8.11	8.67	0.44	0.37	0.42

1 ITM = inorganic chloride trace mineral source of Zn (75 mg/kg DM), Mn (65 mg/kg), and Cu (10 mg/kg) as hydroxychlorides and Co (1 mg/kg) as carbonate and AATM = partial replacement of inorganic trace minerals with AA complexes of trace mineral source of Zn (40 mg/kg), Mn (20 mg/kg), Cu (3.5 mg/kg), and Co glucoheptonate (1 mg/kg).

2 T = treatment and wk = week.

3 Week effect, *P* < 0.0001.

Serum folate concentration from wk −1 to 8 relative to parturition averaged 8.39 (SE: 0.44) ng/mL and was not affected by treatments (*P* = 0.42; Table 1). Regardless of treatments, serum folate concentration significantly decreased from wk −8 to −1 (*P* < 0.0001), plateaued from wk −1 to 2 relative to calving (*P* = 0.81), and then significantly increased thereafter at wk 8 of lactation (*P* < 0.0001). Serum folate concentrations at wk −8 and 8 relative to calving were not different (*P* = 0.34). Other studies have shown that serum or plasma folate concentrations varied according to the stage of lactation (Akins et al., 2013; Duplessis et al., 2017). Serum folate concentrations from wk −8 to 1 relative to calving were similar to the values reported by Akins et al. (2013) for the same time frame but lower than the values reported by others (Duplessis et al., 2017, 2022). Postpartum serum or plasma folate concentrations are variable in the literature. Most studies reported higher plasma folate concentrations at wk 8 of lactation than the current study (Girard and Matte, 1999; Graulet et al., 2007; Duplessis et al., 2022). Moreover, serum folate concentrations at wk 8 of lactation (11.50 [SE: 0.64] ng/mL) in the current study corresponded to the lowest averaged plasma folate concentration reported in the North American cross-sectional study conducted by Duplessis et al. (2020), which corresponded to cows located in California. Serum and plasma folate concentrations are affected by several factors such as parity number, stage of lactation, and the diet, among others (Duplessis et al., 2020). Caution should be taken when interpreting these results because different laboratory analyses were used and could also explain the discrepancy between studies. Under the current state of knowledge, it is unknown if serum or plasma folate concentration is a good indicator of the vitamin status.

Milk yield and DMI were moderately positively related to serum folate concentration, which is in line with the work by Duplessis et al. (2024). Serum ALT and ALP concentrations were moderately positively correlated with serum folate concentration (Spearman correlation coefficient between 0.30 and 0.40, *P* < 0.01; Table 2), whereas serum BHB concentration tended to be negatively correlated with serum folate concentration (Spearman correlation coefficient = −0.22; *P* = 0.07) but the relationship was weak. Alanine aminotransferase is an enzyme involved in gluconeogenesis and, in excess in serum, suggests liver damage (Zaitsev et al., 2020). Alkaline phosphate is also used as a liver damage biomarker (Giannuzzi et al., 2024). In the current experiment, ALT varied from 9 to 19 U/L, which is in the range of normal values reported from healthy cows (Lumsden et al., 1980; Du et al., 2017; Zaitsev et al., 2020), whereas ALP varied from 19 to 64 U/L as similarly obtained by Giannuzzi et al. (2024). However, the averaged ALP concentration for the current study was within the 25th percentile of the study by Giannuzzi et al. (2024). This suggests that cows in the present assessment did not have impaired liver function. In contrast to the current results, in humans, genetically predicted higher serum folate concentrations was reported to be associated with decreased serum ALT concentration but not ALP (Yuan et al., 2022). The links between serum folates and serum ALT, ALP are reported here for the first time and warrant further investigation. Although it was only a tendency in the current assessment, Duplessis et al. (2023) observed a negative relationship between plasma folate and BHB concentrations. The authors concluded that this can be due to an increased use of folates with elevated BHB concentration.

**Table 2:** Spearman rank correlation coefficients between serum folate concentrations and milk yield, DMI, serum biomarkers, and the liver health index^1^

Item	Spearman rank correlation coefficient	*P*-value
Milk yield	0.42	<0.0001
DMI	0.42	<0.0001
BHB	−0.22	0.07
Haptoglobin	−0.11	0.39
Urea	0.02	0.88
Creatinine	0.02	0.90
Albumin	0.13	0.28
Total protein	0.08	0.54
Globulin	−0.01	0.91
Cholesterol	−0.00	0.97
Bilirubin	0.03	0.79
Alanine aminotransferase	0.40	0.0008
Alkaline phosphatase	0.30	0.01
Aspartate aminotransferase	0.06	0.65
Gamma-glutamyl transferase	−0.11	0.41
Liver health index^2^	0.17	0.18

1 Correlations with milk yield used data from wk 1, 2, and 8 postpartum; DMI used data from wk −1, 1, 2, and 8 relative to calving; all other variables used data from wk 1 and 2 relative to calving.

2 Calculated as [(albumin − μ_albumin_)/σ_albumin_] + [(cholesterol − μ_cholesterol_)/σ_cholesterol_] − [(bilirubin − μ_bilirubin_)/σ_bilirubin_], where μ = the overall sampling population mean and σ = the overall sampling population SD (Kerwin et al., 2022).
